# Dietary Modulation of Postoperative Inflammation: Molecular Mechanisms and Implications for Tissue Repair and Healing

**DOI:** 10.3390/ijms27125483

**Published:** 2026-06-17

**Authors:** Charlotta Victoria Siefert, Lara Baticic

**Affiliations:** 1 Faculty of Medicine, University of Rijeka, 51000 Rijeka, Croatia; charlotta.siefert@uniri.hr; 2 Department of Medical Chemistry, Biochemistry and Clinical Chemistry, Faculty of Medicine, University of Rijeka, 51000 Rijeka, Croatia

**Keywords:** postoperative inflammation, diet therapy, inflammation resolution, wound healing, immunometabolism

## Abstract

Postoperative inflammation is a necessary response to surgical injury that supports tissue repair and regeneration. However, successful healing depends not only on the initial inflammatory response but also on its timely resolution. Failure to resolve inflammation can impair wound healing, promote fibrosis, and increase the risk of postoperative complications. Increasing evidence suggests that effective recovery is driven by the transition from inflammation to repair and regenerative processes. Diet plays an important role in this transition, as nutrients not only provide metabolic support but also regulate key pathways involved in inflammation, tissue regeneration, redox balance, and immune function. Omega-3 polyunsaturated fatty acids could serve as precursors for specialized pro-resolving mediators that actively terminate inflammation and may promote macrophage-driven tissue repair. Polyphenols and antioxidant micronutrients modulate NF-κB and Nrf2-dependent signalling, attenuating oxidative amplification of inflammatory cascades. Micronutrients and amino acids further regulate enzymatic processes governing collagen synthesis, angiogenesis, and immune competence. Concurrently, diet-driven preservation of gut barrier integrity limits endotoxin-mediated amplification of systemic inflammatory responses. By targeting interconnected molecular networks, including inflammasome activation, mitochondrial redox signalling, and metabolic programming of immune cells, anti-inflammatory dietary patterns may promote immune resolution rather than immunosuppression. This distinction is particularly relevant in the postoperative setting, where balanced inflammation is required for both host defence and regenerative healing. This review synthesizes current molecular and translational evidence linking dietary modulation to postoperative inflammatory control and tissue regeneration. By integrating insights from immunology, metabolism, and nutritional science, it positions diet as an active, biologically grounded component of perioperative management and highlights future directions for precision nutrition strategies aimed at optimizing surgical recovery.

## 1. Introduction

Surgical injury inevitably triggers a robust inflammatory response that is essential for host defence and tissue repair. In the postoperative setting, this response is initiated by tissue damage, ischemia reperfusion, and cellular stress, leading to the release of damage-associated molecular patterns (DAMPs) such as high-mobility group box 1 protein (HMGB1), extracellular ATP, and mitochondrial DNA [1,2,3]. These signals activate innate immune pathways through pattern-recognition receptors, including Toll-like receptors and nucleotide-binding oligomerization domain–like receptors, resulting in the rapid recruitment of neutrophils and monocytes, production of pro-inflammatory cytokines, and generation of reactive oxygen species [4,5,6]. While this acute inflammatory phase is physiologically necessary, its persistence or dysregulation is a major determinant of postoperative complications, impaired tissue repair, and adverse long-term outcomes [7,8].

Increasing evidence indicates that the clinical problem in postoperative recovery is not excessive inflammation per se, but rather a failure of timely and coordinated inflammatory resolution [9,10]. Under physiological conditions, inflammation is a self-limited process that actively transitions into a resolution phase characterized by clearance of apoptotic cells, reprogramming of macrophages toward reparative phenotypes, restoration of tissue homeostasis, and initiation of regeneration [9,10,11]. Disruption of this transition prolongs inflammatory signalling, sustains oxidative stress, impairs angiogenesis, and promotes fibrosis, compromising wound healing and tissue integrity [12,13,14]. In surgical patients, prolonged inflammatory activity has been linked to delayed epithelialization, impaired microvascular perfusion, increased susceptibility to infection, and suboptimal scar formation [7,12].

At the molecular level, postoperative inflammation is orchestrated by tightly interconnected signalling networks that integrate immune activation, cellular metabolism, and redox balance. Central pathways include nuclear factor kappa B (NF-κB)–dependent transcription of pro-inflammatory cytokines, activation of the NLRP3 inflammasome with subsequent interleukin-1β release, and metabolic reprogramming of immune cells toward glycolytic, pro-inflammatory states [6,13,14,15]. Concurrently, excessive mitochondrial ROS amplify inflammatory signalling and induce collateral tissue injury [16,17]. Resolution of inflammation requires coordinated downregulation of these pathways alongside activation of pro-resolving pathways that promote efferocytosis, restore endothelial function, and support extracellular matrix remodelling [10,11,18].

Dietary factors have emerged as potential critical modulators of these molecular pathways. Beyond their role as energy sources and structural substrates, nutrients may act as signalling molecules that may influence immune cell function, gene transcription, lipid mediator biosynthesis, and mitochondrial activity [19,20,21]. Anti-inflammatory dietary patterns, characterized by high intake of omega-3 polyunsaturated fatty acids, plant-derived polyphenols, antioxidant vitamins, and essential trace elements, have been shown to suppress pro-inflammatory signalling while promoting pathways associated with immune resolution [19,22,23]. Notably, omega-3 fatty acids serve as precursors for specialized pro-resolving mediators, including resolvins, protectins, and maresins, which actively terminate inflammation without compromising host defence [10,18,24]. Polyphenols and antioxidant micronutrients further modulate transcriptional and redox-sensitive pathways, attenuating NF-κB activation and enhancing endogenous antioxidant capacity [20,25].

**Figure 1:**
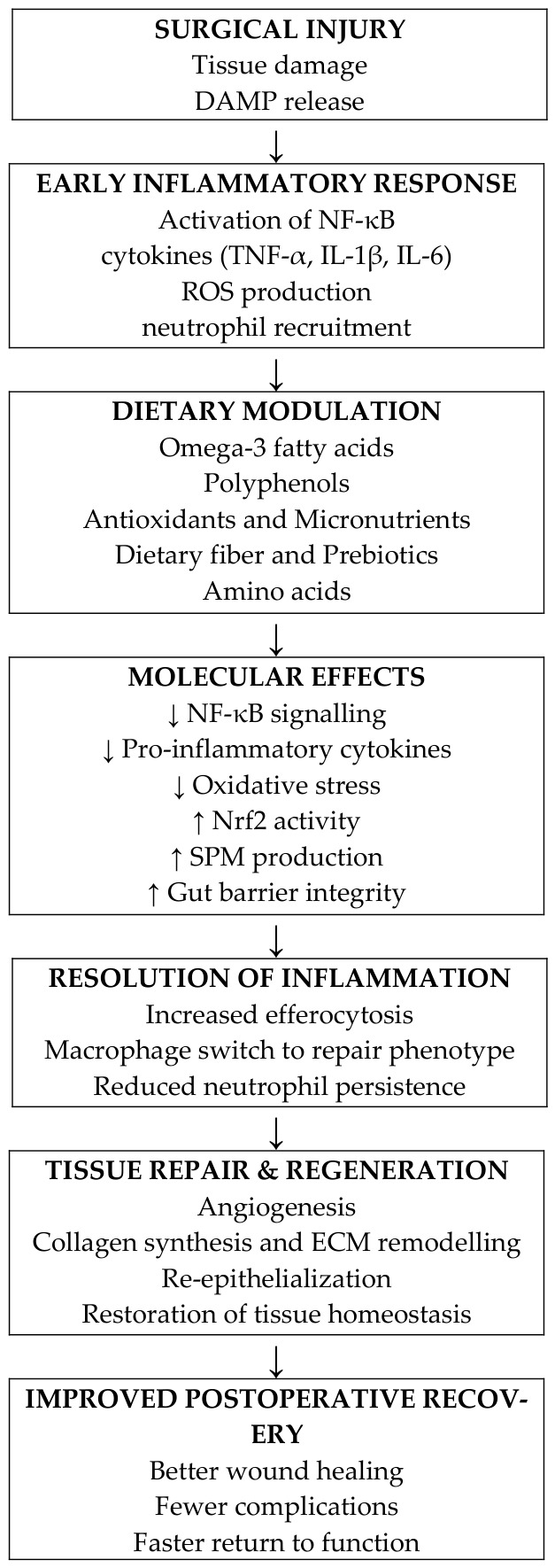
Simplified overview of the proposed effects of dietary factors on postoperative inflammation and recovery. Following surgical injury, tissue damage and release of damage-associated molecular patterns (DAMPs) initiate an early inflammatory response characterized by activation of NF-κB signalling, cytokine production, oxidative stress, and neutrophil recruitment. Dietary components, including omega-3 fatty acids, polyphenols, antioxidants, micronutrients, dietary fiber, prebiotics, and amino acids, may modulate key molecular pathways involved in inflammation resolution. These effects may contribute to reduced inflammatory signalling and oxidative stress, enhanced specialized pro-resolving mediator (SPM) production, improved gut barrier integrity, and activation of repair-associated immune responses. Collectively, these processes may support inflammation resolution, tissue repair and regeneration, and improved postoperative recovery. The figure represents a simplified schematic overview and does not capture the full complexity, temporal overlap, or feedback interactions of the biological processes involved. Abbreviations: DAMPs, damage-associated molecular patterns; ECM, extracellular matrix; IL, interleukin; NF-κB, nuclear factor kappa B; Nrf2, nuclear factor erythroid 2-related factor 2; ROS, reactive oxygen species; SPMs, specialized pro-resolving mediators; TNF-α, tumor necrosis factor alpha.

The relevance of dietary modulation is particularly pronounced in the postoperative context, where metabolic stress, transient immune dysregulation, and gut barrier dysfunction unite to amplify systemic inflammation [26,27]. Surgical stress is frequently accompanied by alterations in intestinal permeability and microbiome composition, facilitating translocation of microbial products that further activate inflammatory signalling cascades [28,29]. Nutritional composition may directly influence these gut–immune interactions, shaping endotoxemia, cytokine production, and immune cell trafficking [30,31]. Thus, diet represents a systemic lever through which inflammatory tone and resolution capacity can be modulated during the critical perioperative period. To facilitate understanding of the complex molecular interactions discussed in the following sections, a simplified overview of the relationship between dietary factors, inflammation resolution, tissue repair, and postoperative recovery is presented in Figure 1.

Despite growing recognition of the relationship between nutrition and surgical outcomes, much of the existing literature remains focused on clinical endpoints such as infection rates, length of hospital stays, or wound closure time, with limited integration of underlying molecular mechanisms [32,33]. A mechanistic synthesis is therefore required to bridge immunology, metabolism, and nutritional science, and to clarify how dietary interventions reshape postoperative inflammatory networks at the cellular and molecular levels.

The aim of this review was to examine the molecular mechanisms by which dietary factors regulate postoperative inflammation, with particular emphasis on pathways that may promote immune resolution and tissue regeneration. We focus on diet-induced modulation of inflammatory signalling, lipid mediator biosynthesis, immunometabolism, redox balance, and gut–immune interactions and discuss how these processes collectively influence tissue repair following surgical injury.

## 2. Methods

### 2.1. Literature Search Strategy

A structured literature search was conducted to identify relevant studies examining the role of dietary factors in the modulation of postoperative inflammation, immune resolution, and tissue regeneration. Searches were performed in PubMed and Web of Science for articles published up to February 2026, using combinations of the following terms: (“postoperative inflammation” OR “surgical stress”) AND (“diet” OR “nutrition” OR “omega-3 fatty acids” OR “polyphenols”) AND (“immune resolution” OR “specialized pro-resolving mediators” OR “NF-κB” OR “NLRP3 inflammasome” OR “immunometabolism” OR “oxidative stress” OR “gut–immune axis” OR “wound healing” OR “tissue regeneration”). In addition, reference lists of relevant articles were manually screened to identify further eligible studies. Priority was given to peer-reviewed mechanistic, translational, and clinically relevant studies published in established scientific journals.

### 2.2. Study Selection and Eligibility

Studies were screened based on titles and abstracts, followed by full-text evaluation for eligibility. Eligible studies included original research articles, experimental studies, clinical trials, observational studies, and relevant reviews addressing dietary modulation of inflammation, immune signalling, metabolic regulation, and tissue repair processes in the context of surgical injury or related inflammatory conditions. Particular emphasis was placed on studies investigating molecular pathways such as NF-κB signalling, inflammasome activation, immunometabolic reprogramming, redox regulation, and pro-resolving lipid mediator biology. Exclusion criteria included non-English publications, case reports, and studies not directly relevant to postoperative or sterile inflammation and its nutritional modulation. Study selection was guided by relevance to the research question and conceptual contribution.

### 2.3. Data Synthesis and Methodological Approach

This review was designed as a narrative synthesis to integrate heterogeneous evidence across molecular, immunological, metabolic, and clinical domains. Given the substantial heterogeneity in study designs, experimental models, and reported outcomes, a quantitative meta-analysis was not feasible. Where available, evidence from human studies and clinical investigations was prioritized, while mechanistic and preclinical studies were included to provide biological context and support the interpretation of clinical observations. Potential sources of bias, including study design limitations, experimental variability, and translational gaps, were considered qualitatively during data interpretation. The aim was to provide an integrative and mechanistically grounded synthesis of current evidence, to identify key regulatory pathways linking diet and postoperative inflammation, and to highlight emerging concepts relevant to immune resolution and tissue regeneration in the perioperative setting. As this review was designed as a narrative synthesis rather than a systematic review, the number of screened and included studies was not formally tracked.

## 3. Molecular Architecture of Postoperative Inflammation and Immune Resolution

Surgical trauma initiates a multilayered molecular response that extends beyond a simple inflammatory cascade. Rather, postoperative inflammation emerges from the coordinated interaction of tissue injury detection, intracellular danger signalling, leukocyte recruitment, endothelial activation, metabolic adaptation, and resolution programming. The biological outcome of this response is highly context-dependent. When tightly controlled, it enables microbial defence, removal of necrotic material, restoration of perfusion, and tissue regeneration. When excessive, prolonged, or poorly resolved, it promotes endothelial dysfunction, oxidative injury, persistent leukocyte activation, aberrant matrix remodelling, fibrosis, and delayed wound healing [1,2,3,34,35].

Postoperative inflammation is a dynamic process rather than a series of separate steps. Early signals released after tissue injury activate immune and metabolic pathways that determine the strength of the inflammatory response and influence subsequent healing and recovery [4,6,13,15]. These early signals then determine the efficiency of the later transition toward efferocytosis, immune silencing, tissue remodelling, and restoration of homeostasis. Accordingly, the decisive biological question is not whether inflammation occurs, but whether the injured tissue can execute an efficient switch from inflammatory amplification to programmed resolution [9,10,11,18].

**Figure 2:**
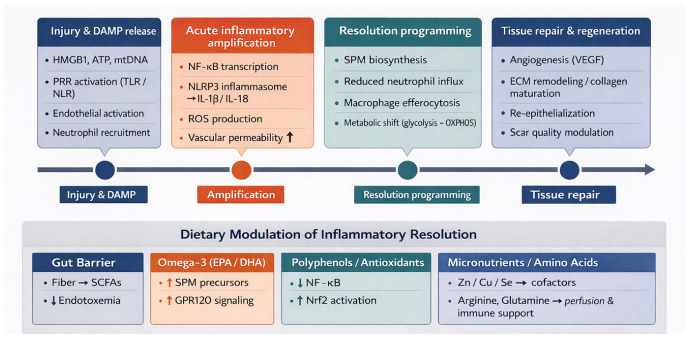
Molecular timeline of postoperative inflammation and resolution and dietary modulators influencing each phase. The figure provides a simplified schematic overview of the temporal progression from surgical injury and inflammatory activation to resolution, tissue repair, and recovery. Dietary factors may influence key molecular pathways involved in these processes. The phases shown are highly interconnected and may overlap temporally in vivo. Abbreviations: ATP, adenosine triphosphate; DAMPs, damage-associated molecular patterns; DHA, docosahexaenoic acid; ECM, extracellular matrix; EPA, eicosapentaenoic acid; HMGB1, high-mobility group box 1; IL, interleukin; mtDNA, mitochondrial DNA; NF-κB, nuclear factor kappa B; NLRP3, NOD-like receptor family pyrin domain-containing 3; OXPHOS, oxidative phosphorylation; ROS, reactive oxygen species; SPMs, specialized pro-resolving mediators; TLR, Toll-like receptor; VEGF, vascular endothelial growth factor. Created using BioRender.com (BioRender, Toronto, ON, Canada).

Under physiological conditions, postoperative inflammation follows a coordinated temporal sequence. Within minutes to hours after surgical injury, tissue damage triggers the release of DAMPs, activation of innate immune pathways, and recruitment of neutrophils. During the first 24–72 h, inflammatory signalling reaches its peak. This is characterized by cytokine production, inflammasome activation, and oxidative stress. Resolution processes are subsequently initiated, with reduced neutrophil influx, enhanced efferocytosis, macrophage reprogramming toward reparative phenotypes, and increased production of specialized pro-resolving mediators. Over the following days to weeks, these processes support angiogenesis, extracellular matrix remodeling, re-epithelialization, and restoration of tissue homeostasis. The coordinated interaction between inflammatory amplification, resolution programming, and diet-dependent regulatory mechanisms is summarized in Figure 2.

### 3.1. Initiation of Sterile Inflammation After Surgical Injury

In the absence of infection, surgical trauma induces sterile inflammation through the release of damage-associated molecular patterns from disrupted, ischemic, or stressed cells. These include high-mobility group box 1 protein, ATP, uric acid, heat-shock proteins, extracellular matrix fragments, mitochondrial DNA, N-formyl peptides, and cardiolipin [1,2,3,36]. Mitochondrial components are particularly potent activators of the immune system because they share structural similarities with bacteria due to their evolutionary origin [17,36,37]. These endogenous danger signals are detected by pattern-recognition receptors on tissue-resident macrophages, infiltrating monocytes, dendritic cells, neutrophils, endothelial cells, and stromal cells. Toll-like receptors, nucleotide-binding oligomerization domain-like receptors, receptor for advanced glycation end products, and purinergic receptors together form an integrated danger-sensing network that translates tissue injury into inflammatory gene expression [4,6,13,37]. TLR4-mediated recognition of HMGB1 and related ligands is especially relevant, as it activates both MyD88- and TRIF-dependent pathways and induces rapid transcription of TNF-α, IL-6, pro-IL-1β, chemokines, adhesion molecules, and enzymes involved in eicosanoid synthesis [4,6,38].

At the same time, blood vessel lining cells (endothelial cells) become activated in response to DAMPs, cytokines, and reactive oxygen species (ROS). This promotes the recruitment and movement of immune cells into injured tissues and contributes to changes in microvascular function [7,34,39]. This endothelial response is essential for immune cell recruitment, yet when excessive it contributes to edema, impaired tissue oxygenation, and ischemia-reperfusion injury, all of which can aggravate postoperative tissue damage [7,34,39].

In the early phase of inflammation, neutrophils are the first immune cells to arrive at the site of injury [39]. They clear debris and provide antimicrobial protection, but they also release proteases, myeloperoxidase, and reactive oxygen species that can injure viable tissue when neutrophil persistence is prolonged [39]. Monocytes subsequently enter the wound bed and differentiate into macrophages whose phenotype is shaped by local cytokines, metabolites, oxygen tension, and engulfment signals. This initial leukocyte architecture sets the stage for the later balance between persistent inflammation and regenerative repair [18,35,40,41].

### 3.2. NF-κB Signalling as a Central Regulatory Hub

NF-κB occupies a central position within the molecular architecture of postoperative inflammation because it integrates danger recognition, cytokine feedback, oxidative stress, and metabolic inputs into a unified transcriptional response. Activation of canonical NF-κB signalling downstream of TLRs, TNF receptors, IL-1 receptors, and redox-sensitive kinases induces expression of numerous inflammatory mediators, including IL-6, TNF-α, IL-1β, CXCL8, COX-2, inducible nitric oxide synthase, matrix metalloproteinases, and anti-apoptotic factors [6,38].

This broad activation is important in the early phase of healing because it helps recruit immune cells, protect against infection, and remove damaged tissue [6,38]. However, persistent NF-κB activity becomes maladaptive. Sustained cytokine production maintains endothelial activation, prolongs neutrophil influx, enhances proteolytic tissue destruction, and interferes with the transition from inflammation to proliferation. Excessive NF-κB signalling has been associated with impaired keratinocyte migration, reduced fibroblast efficiency, delayed angiogenic maturation, and dysregulated extracellular matrix remodelling [34,35,42,43].

NF-κB is also linked to cellular energy use. In activated macrophages, increased glycolysis provides the energy and metabolic signals needed to support inflammatory responses and the production of inflammatory mediators [15,44]. Thus, NF-κB functions not only as a transcription factor but also as part of a broader immunometabolic state. Recent perioperative immunometabolism literature further supports the concept that surgical stress reshapes leukocyte metabolism in ways that directly modulate inflammatory signalling and recovery trajectories [40].

An additional level of complexity arises from the interaction between NF-κB and redox biology. Oxidative stress amplifies NF-κB activation through redox-sensitive kinase systems, while NF-κB itself induces expression of enzymes that further influence ROS and reactive nitrogen species generation. This creates a cycle in which oxidative stress and inflammation amplify each other, potentially prolonging tissue damage unless balanced by antioxidant and pro-resolving mechanisms [16,17,25].

### 3.3. Inflammasome Activation and Amplification of Inflammatory Signalling

The inflammasome represents a second major regulatory axis of postoperative inflammation. Among the inflammasome family members, NLRP3 is most closely linked to sterile tissue injury. Its activation typically follows a two-signal model. A priming signal, often provided by TLR- or cytokine-induced NF-κB activation, increases transcription of NLRP3 and pro-IL-1β. A second activation signal is then generated by mitochondrial dysfunction, ROS accumulation, ion fluxes, lysosomal destabilization, ATP-mediated P2X7 signalling, or other forms of intracellular stress [13,14,45,46].

Once assembled, the NLRP3 inflammasome activates caspase-1, enabling cleavage of pro-IL-1β and pro-IL-18 into their mature forms and promoting gasdermin D pore formation, cytokine release, and pyroptotic signalling. In the postoperative setting, this pathway intensifies local inflammation by enhancing neutrophil recruitment, endothelial activation, vascular leakage, and protease release [13,45,46,47]. Although transient inflammasome activation may be necessary for early host defence and damage containment, excessive or prolonged activation can markedly impair regenerative healing.

Current research suggests that prolonged inflammasome activation may contribute to persistent inflammation and impaired tissue healing. High levels of IL-1β can promote tissue damage, delay the transition of macrophages to repair-promoting states, and favor fibrosis rather than regeneration [47,48]. This process may be particularly relevant after surgery, where ischemia-reperfusion injury, mitochondrial dysfunction, and metabolic stress can further enhance inflammasome activation [13,14,45,46]. Importantly, inflammasome regulation is not independent of broader cellular housekeeping systems. Autophagy and selective mitophagy normally limit inflammasome activation by removing damaged mitochondria, reducing mitochondrial ROS, and preventing accumulation of mitochondrial DNA and other pro-inflammatory ligands in the cytosol [41,46,49]. When autophagic flux is impaired, inflammasome activation becomes more persistent and difficult to resolve. This makes the NLRP3-autophagy-mitochondria axis a particularly relevant mechanistic bridge between tissue injury, metabolism, and postoperative immune dysfunction.

### 3.4. Immunometabolic Reprogramming During Postoperative Inflammation

Postoperative inflammation is inseparable from metabolic adaptation. Immune cells do not merely consume energy while performing inflammatory tasks; instead, their metabolic configuration actively determines phenotype and function. Neutrophils and classically activated macrophages preferentially rely on aerobic glycolysis, a program that supports rapid ATP production, pentose phosphate pathway flux, ROS generation, and synthesis of inflammatory mediators [15,40,44,50]. This metabolic state enables immediate defence but also stabilizes a pro-inflammatory cellular identity.

Several metabolites generated during this state act as signalling intermediates. Succinate, for example, may promote inflammatory activation through HIF-1α-dependent mechanisms and reinforces IL-1β production [44]. Citrate supports lipid mediator synthesis and nitric oxide generation, whereas lactate accumulation contributes to local acidosis and modifies immune cell behaviour. Together, these metabolic changes create an inflammatory microenvironment that extends beyond conventional cytokine signalling.

Resolution of inflammation requires a distinct metabolic shift. Reparative macrophages increasingly depend on mitochondrial oxidative phosphorylation, fatty acid oxidation, lysosomal lipid metabolism, and efficient mitochondrial quality control [15,40,41,50,51]. This transition supports efferocytosis, growth factor production, matrix remodelling, and secretion of mediators such as TGF-β and VEGF. Failure to undergo this metabolic reprogramming traps macrophages in a pro-inflammatory state and delays wound progression toward proliferation and remodelling.

Key regulatory nodes in this transition include AMPK, mTOR, PPARs, sirtuins, and hypoxia-responsive pathways [40,50,52]. Their activity is influenced by nutrient availability, oxygen tension, mitochondrial integrity, and intracellular redox balance. Thus, postoperative inflammation should be viewed as an immunometabolic process in which nutrient-sensitive signalling pathways determine whether immune cells remain destructive or become reparative.

Macrophage biology is especially important in this regard. Recent research suggests that macrophages do not exist as only two distinct types (M1 and M2), but rather as a range of dynamic states influenced by the local environment. These states are shaped by cytokines, metabolites, tissue signals, and epigenetic factors [41,53]. Therefore, successful postoperative healing depends not only on the recruitment of macrophages but also on their timely transition toward tissue repair and regeneration.

### 3.5. Mitochondrial Function, Redox Signalling, and Intracellular Quality Control

Mitochondria are central regulators of postoperative inflammation because they integrate metabolism, ROS generation, apoptosis, innate immune activation, and resolution-phase reprogramming. During surgical stress and ischemia-reperfusion, mitochondria undergo membrane depolarization, electron transport chain dysfunction, calcium overload, and altered dynamics. These disturbances increase mitochondrial ROS production and facilitate the release of mitochondrial DAMPs, thereby amplifying inflammatory signalling [16,17,41,49].

Mitochondrial ROS play a dual role. At physiological concentrations, they act as signalling mediators required for antimicrobial defence and adaptive cellular responses. At excessive levels, they damage lipids, proteins, and nucleic acids, disrupt endothelial integrity, and promote inflammasome activation. This distinction is highly relevant in the postoperative context, where controlled redox signalling supports healing, while oxidative overload drives collateral tissue injury and delayed recovery [16,17,54].

Mitochondrial quality control mechanisms such as fusion, fission, mitophagy, and proteostatic surveillance are therefore essential for containing inflammatory damage. Efficient mitophagy selectively removes dysfunctional mitochondria before they become major sources of ROS and mtDNA release. Conversely, impaired mitochondrial quality control prolongs sterile inflammation by sustaining inflammasome activation and activating cytosolic innate immune pathways such as cGAS-STING [41,49]. These findings broaden the conventional concept of postoperative inflammation by showing that intracellular organelle quality control is itself an immune-regulatory process.

Because mitochondrial function is tightly connected to substrate supply, antioxidant defence, membrane lipid composition, and micronutrient status, it represents a major point of interaction between nutrition and inflammation. Nutritional factors do not simply attenuate cytokines downstream; they can alter the very intracellular conditions that determine whether mitochondria may promote resolution or perpetuate inflammatory damage.

Mitochondrial function is also highly dependent on adequate B-vitamin availability. B vitamins serve as essential cofactors in mitochondrial energy metabolism, including the tricarboxylic acid cycle, oxidative phosphorylation, and one-carbon metabolism. Emerging evidence suggests that common genetic polymorphisms affecting folate and vitamin B12 metabolism may influence mitochondrial efficiency, redox balance, and inflammatory responses. Although a detailed discussion of B-vitamin metabolism is beyond the scope of this review, these nutrients represent an additional link between diet, mitochondrial function, and postoperative recovery [16,17,41,49].

### 3.6. Efferocytosis and the Transition to Reparative Immunity

Successful resolution of postoperative inflammation depends on the efficient removal of dead neutrophils and cellular debris, a process known as efferocytosis. This is an important step in wound healing because it helps macrophages switch from a pro-inflammatory to a repair-promoting state [9,11,18]. During this process, macrophages recognize and engulf dying cells, which reduces inflammation and supports tissue repair and regeneration [18,55,56].

Efferocytosis has effects that go far beyond debris removal. It suppresses production of pro-inflammatory cytokines, induces secretion of IL-10 and TGF-β, promotes lipid mediator class switching, and drives metabolic reprogramming toward oxidative phosphorylation and fatty acid utilization [18,51,55,56]. In this way, efferocytosis acts as a mechanistic switch from inflammatory amplification to tissue reconstruction. Impaired efferocytosis, by contrast, leads to secondary necrosis of apoptotic cells, renewed DAMP release, sustained cytokine production, and poor healing outcomes.

Recent transcriptional analyses of wound macrophages show that efferocytosis is associated with a distinct pro-repair gene program, underscoring that resolution is an actively encoded cellular state rather than a passive decline in inflammation [56]. These findings also explain why persistent neutrophil accumulation is detrimental not simply because neutrophils are injurious, but because unresolved apoptotic cell clearance prevents the macrophage transition required for regenerative repair.

### 3.7. Active Resolution of Inflammation as a Programmed Biological Process

Resolution is now recognized as a highly organized biological program rather than the spontaneous disappearance of inflammatory stimuli. Specialized pro-resolving mediators generated from omega-3 polyunsaturated fatty acids are central to this phase. These mediators, including resolvins, protectins, maresins, and conjugates in tissue regeneration, actively terminate neutrophil influx, enhance macrophage efferocytosis, reduce inflammatory cytokine production, and stimulate tissue regeneration without causing broad immunosuppression [9,18,24,57,58,59].

This distinction is especially important in postoperative patients. The objective is not to eliminate inflammation altogether, because early inflammatory activation is necessary for host defence and wound debridement. Instead, successful recovery requires an efficient lipid mediator class switch from prostaglandin- and leukotriene-dominated inflammatory signalling toward pro-resolving mediator production [9,10,18,24]. When this switch fails, tissues remain trapped in a state of persistent leukocyte activity, oxidative injury, and is associated with non-resolving inflammation and impaired or incomplete tissue healing.

Emerging work further suggests that pro-resolving mediators influence macrophage polarization, epithelial repair, angiogenesis, and matrix organization at transcriptional and metabolic levels [58,59]. Thus, resolution biology forms the conceptual bridge between dietary lipid composition and clinical wound healing outcomes.

### 3.8. Epigenetic Regulation of Inflammatory Resolution

An additional regulatory layer is provided by epigenetic control. Postoperative inflammation is not governed solely by receptor signalling and metabolite flux, but also by chromatin accessibility, histone acetylation, histone methylation, DNA methylation, and non-coding RNA networks that determine which inflammatory or reparative genes can be expressed [49,60,61,62]. These mechanisms are dynamic and responsive to the tissue microenvironment.

In wound healing, histone modifications appear to regulate both the intensity and the duration of inflammation, particularly by influencing neutrophil extracellular trap formation in early phases and macrophage reprogramming during later phases [60,61]. Persistent pathological microenvironments can lock immune cells into pro-inflammatory transcriptional states, thereby promoting chronic non-resolving inflammation. Conversely, appropriate epigenetic remodelling supports the normal progression from inflammatory activation to proliferation and remodelling.

This is highly relevant for nutritional physiology because several diet-derived molecules and microbiome-derived metabolites affect epigenetic machinery. Short-chain fatty acids such as butyrate can inhibit histone deacetylases, modify acetyl-CoA availability, activate G-protein-coupled receptors, and thereby influence inflammatory transcriptional programs [31,62,63]. Polyphenols and fatty acids likewise affect epigenetic regulation of immune genes. These observations provide a mechanistic explanation for how dietary patterns can shape postoperative inflammatory trajectories beyond immediate receptor-level effects.

### 3.9. Integrated View of Postoperative Inflammation as a Resolution-Dependent Network

Taken together, postoperative inflammation can be viewed as a complex and interconnected network involving danger sensing, inflammatory signalling, immunometabolic changes, mitochondrial function, efferocytosis, specialized pro-resolving mediators, and epigenetic regulation [4,6,9,10,11,41,49,60]. These processes do not act independently but continuously influence one another. For example, mitochondrial dysfunction may enhance inflammasome activation, while metabolic changes affect immune cell behavior and inflammation resolution [15,41,46,49,50]. The coordinated interaction of these pathways ultimately determines whether healing progresses toward tissue repair and recovery or toward persistent inflammation and complications [9,10,11,18,34,35]. This network perspective is particularly important in the postoperative setting, where surgical injury simultaneously affects immune function, metabolism, blood flow, and tissue structure [7,15,34,40]. Recovery depends not only on the initial inflammatory response but also on the ability to transition efficiently from inflammation to resolution and tissue repair [9,10,11,18]. Understanding these interconnected mechanisms provides a biological basis for nutritional strategies that may help support postoperative healing and recovery.

## 4. Dietary Modulation of Inflammatory Signalling and Immune Resolution

### 4.1. Omega-3 Fatty Acids and Specialized Pro-Resolving Mediators

Omega-3 polyunsaturated fatty acids (PUFAs) represent one of the most extensively studied dietary modulators of inflammation and immune resolution. The principal bioactive omega-3 fatty acids in human physiology, eicosapentaenoic acid (EPA) and docosahexaenoic acid (DHA), exert their effects not merely by the possibility to suppress inflammatory signalling but also by the ability to contribute to reprogramming the inflammatory response toward resolution and tissue repair. This distinction is of relevance in the postoperative setting, where effective resolution rather than broad immunosuppression determines regenerative success.

**Figure 3:**
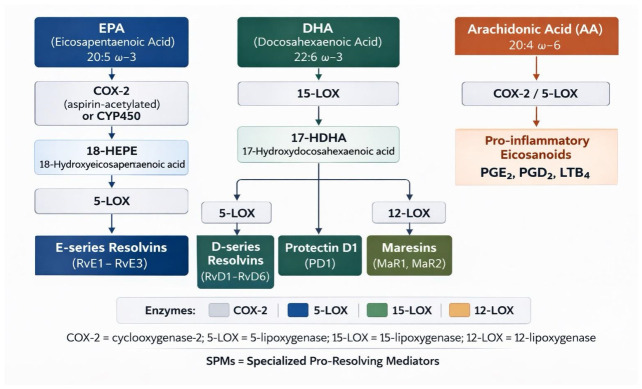
Biosynthesis of Specialized Pro-Resolving Mediators from Omega-3 Fatty Acids. EPA and DHA serve as precursors for specialized pro-resolving mediators (SPMs), including resolvins, protectins, and maresins, through cyclooxygenase (COX) and lipoxygenase (LOX)-dependent pathways. These mediators may contribute to the resolution of inflammation and support tissue repair processes. Abbreviations: AA, arachidonic acid; COX, cyclooxygenase; DHA, docosahexaenoic acid; EPA, eicosapentaenoic acid; HEPE, hydroxyeicosapentaenoic acid; HDHA, hydroxydocosahexaenoic acid; LOX, lipoxygenase; PG, prostaglandin; LTB4, leukotriene B4; SPMs, specialized pro-resolving mediators. Created using BioRender.com (BioRender, Toronto, ON, Canada).

At the membrane level, EPA and DHA incorporate into phospholipid bilayers of immune and endothelial cells, displacing arachidonic acid (AA) and altering the substrate availability for cyclooxygenase (COX) and lipoxygenase (LOX) enzymes [19,64]. This shift results in reduced synthesis of pro-inflammatory eicosanoids such as prostaglandin E_2_ and leukotriene B_4_, while favouring the generation of less inflammatory derivatives, including prostaglandin E_3_ and leukotriene B_5_ [19,65]. However, the most critical consequence of omega-3 incorporation is the enzymatic conversion of EPA and DHA into specialized pro-resolving mediators (SPMs), a distinct class of lipid autacoids that actively orchestrate the termination of inflammation. The enzymatic pathways generating E-series and D-series resolvins from EPA and DHA are illustrated in Figure 3.

SPMs comprise several families, including E-series resolvins (derived from EPA), D-series resolvins, protectins, and maresins (derived from DHA). These mediators are synthesized locally at sites of inflammation through tightly regulated COX-2 and LOX pathways and exert potent biological effects at nanomolar concentrations [10,18,24,57]. In contrast to classical anti-inflammatory agents, SPMs do not inhibit immune activation globally; instead, they may promote resolution by limiting further neutrophil recruitment, enhancing macrophage efferocytosis of apoptotic cells, and restoring tissue homeostasis [10,66].

Mechanistically, SPMs signal through specific G-protein-coupled receptors expressed on immune cells, including ChemR23, ALX/FPR2, and GPR32. Activation of these receptors suppresses NF-κB signalling, downregulates inflammasome activation, and induces transcriptional programs associated with reparative macrophage phenotypes [18,24,67]. In macrophages, SPM signalling facilitates the metabolic transition from glycolysis toward oxidative phosphorylation and fatty acid oxidation, a shift that is essential for resolution-phase functions such as debris clearance, angiogenic support, and extracellular matrix remodelling [15,50,51].

In the postoperative context, these molecular actions have direct relevance. Surgical injury is associated with excessive neutrophil accumulation, oxidative stress, and endothelial dysfunction, all of which can compromise microvascular perfusion and tissue viability. Experimental models of tissue injury demonstrate that administration of resolvins or DHA-derived mediators reduces neutrophil infiltration, limits ROS-mediated collateral damage, and accelerates wound closure [66,68]. Importantly, these effects occur without impairing antimicrobial defence, underscoring the resolution-promoting rather than immunosuppressive nature of omega-3–derived mediators.

Omega-3 fatty acids can also modulate inflammatory signalling upstream of SPM biosynthesis through receptor-mediated pathways (e.g., GPR120/FFAR4), which repress macrophage-driven inflammatory signalling and attenuate cytokine production [69]. In parallel, omega-3s improve endothelial nitric oxide bioavailability, enhance microcirculatory flow, and reduce platelet aggregation which are mechanisms that are particularly important in reconstructive and microvascular surgery, where perfusion and thrombosis risk critically influence outcomes [70,71].

Clinical and translational studies support these molecular findings. Perioperative omega-3 supplementation has been associated with reductions in circulating inflammatory markers, including C-reactive protein and IL-6, as well as improved immune competence and reduced postoperative complications in surgical patients [33,72]. While most clinical studies report outcomes at the systemic level, emerging lipidomic analyses indicate that dietary omega-3 intake may increase tissue and plasma concentrations of SPMs, linking nutritional exposure to molecular resolution pathways [57,73].

Collectively, omega-3 fatty acids function as biochemical precursors and signalling modulators that may contribute to the regulation of postoperative inflammation toward resolution. By simultaneously attenuating pro-inflammatory signalling, promoting SPM biosynthesis, and supporting immunometabolic transitions, omega-3s occupy a central position in dietary strategies aimed at enhancing immune resolution and tissue regeneration after surgical injury. However, although SPM biology represents a promising therapeutic concept, clinical studies evaluating omega-3-based interventions in surgical patients have produced heterogeneous results, and further research is needed to establish their clinical efficacy and optimal implementation.

### 4.2. Polyphenols, Antioxidants, and Redox-Sensitive Regulation of Inflammation

Oxidative stress represents a central amplifier of postoperative inflammation and a critical determinant of tissue injury following surgical trauma. The inflammatory response to surgery is accompanied by excessive generation of reactive oxygen species (ROS) from activated neutrophils, macrophages, and dysfunctional mitochondria. While physiological levels of ROS function as signalling molecules in host defence and tissue repair, excessive or prolonged oxidative stress damages cellular lipids, proteins, and nucleic acids, disrupts redox sensitive signaling pathways, and perpetuates inflammatory activation [16,17,54]. Dietary polyphenols and antioxidant micronutrients exert regulatory effects at this redox–inflammation interface, thereby influencing immune resolution and regenerative capacity.

Polyphenols constitute a diverse class of plant-derived bioactive compounds, including flavonoids, phenolic acids, stilbenes, and curcuminoids, that modulate inflammation primarily through effects on transcriptional control and intracellular redox balance. At the molecular level, many polyphenols inhibit activation of nuclear factor kappa B (NF-κB) by suppressing upstream kinases, stabilizing inhibitory IκB proteins, and reducing oxidative activation of redox-sensitive signalling nodes [20,74,75]. This results in decreased transcription of pro-inflammatory cytokines, chemokines, and enzymes such as cyclooxygenase-2 and inducible nitric oxide synthase, thereby attenuating the magnitude and duration of inflammatory responses.

In parallel, polyphenols activate the nuclear factor erythroid 2–related factor 2 (Nrf2) pathway, a master regulator of cellular antioxidant defence. Nrf2 activation induces expression of genes encoding endogenous antioxidant enzymes, including superoxide dismutase, catalase, glutathione peroxidase, and heme oxygenase-1, enhancing the cell’s capacity to neutralize ROS and restore redox homeostasis [25,76]. In the postoperative setting, upregulation of Nrf2-dependent pathways protects endothelial cells, fibroblasts, and keratinocytes from oxidative injury, supporting angiogenesis, collagen synthesis, and epithelial regeneration. In addition to polyphenols, sulforaphane, an isothiocyanate derived from cruciferous vegetables such as broccoli, is recognized as one of the most potent dietary activators of the Nrf2 pathway. Sulforaphane promotes the expression of antioxidant and cytoprotective genes involved in redox regulation, cellular stress responses, and detoxification processes. Through activation of Nrf2-dependent signalling, sulforaphane has been shown to attenuate oxidative stress, modulate inflammatory responses, and support tissue repair, highlighting its potential relevance in postoperative recovery and inflammation resolution [25,34,76,77].

Antioxidant vitamins and trace elements act synergistically with polyphenols to reinforce redox control. Vitamin C (ascorbic acid) functions as a potent water-soluble antioxidant and an essential cofactor for prolyl and lysyl hydroxylases involved in collagen maturation. By scavenging free radicals and regenerating oxidized vitamin E, vitamin C preserves extracellular matrix integrity and supports tensile strength during wound repair [78,79]. Vitamin E (α-tocopherol), a lipid-soluble antioxidant, protects polyunsaturated fatty acids within cell membranes from lipid peroxidation, stabilizing immune and endothelial cell membranes under conditions of ischemia–reperfusion stress [80].

Trace elements such as selenium, zinc, and copper further contribute to redox regulation by serving as cofactors for antioxidant and repair enzymes. Selenium is an integral component of glutathione peroxidase, a key enzyme responsible for detoxifying hydrogen peroxide and lipid hydroperoxides. Zinc stabilizes cellular membranes and regulates metalloproteinases involved in extracellular matrix remodelling, while copper supports lysyl oxidase–mediated collagen cross-linking and angiogenesis [78,81,82]. Deficiencies in these micronutrients exacerbate oxidative damage, prolong inflammatory signalling, and impair wound healing, highlighting their relevance in the postoperative context.

Beyond direct antioxidant effects, redox modulation influences immune cell phenotype and function. Excessive ROS generation may promote pro-inflammatory macrophage polarization and inflammasome activation, whereas restoration of redox balance facilitates macrophage transition toward reparative, resolution-phase phenotypes [48,50,83]. Polyphenol-induced suppression of ROS and activation of Nrf2 signalling have been shown to inhibit NLRP3 inflammasome assembly and reduce interleukin-1β production, linking antioxidant activity directly to attenuation of inflammasome-driven inflammation [77,84].

Importantly, antioxidant and polyphenol actions do not abolish inflammatory signalling but recalibrate it, preserving necessary immune functions while preventing collateral tissue damage. This regulatory profile is particularly advantageous in the postoperative setting, where excessive suppression of inflammation can impair host defence and increase infection risk. By maintaining redox equilibrium and modulating transcriptional programs, dietary antioxidants support a controlled inflammatory response that transitions efficiently into resolution and tissue regeneration.

Clinical and translational studies provide indirect support for these molecular mechanisms. Diets rich in polyphenols and antioxidant micronutrients are associated with lower postoperative levels of inflammatory markers, reduced oxidative stress indices, and improved wound-healing parameters across surgical populations [22,32,85]. Although direct measurement of tissue-level redox signalling remains limited in clinical studies, emerging biomarker and metabolomic analyses suggest that nutritional antioxidant status significantly influences postoperative inflammatory trajectories.

In summary, polyphenols and antioxidant micronutrients act as critical regulators of redox-sensitive inflammatory signalling, linking dietary composition to immune resolution and tissue repair. Through coordinated inhibition of NF-κB, activation of Nrf2-dependent antioxidant programs, and suppression of oxidative amplification of inflammasome activity, these dietary components complement omega-3–derived pro-resolving pathways and contribute to an integrated, resolution-oriented inflammatory response following surgical injury.

### 4.3. Micronutrients, Amino Acids, and Enzymatic Control of Tissue Regeneration

Micronutrients and conditionally essential amino acids serve as indispensable cofactors and substrates for enzymatic reactions governing immune competence, angiogenesis, and extracellular matrix remodelling during postoperative recovery. Unlike macronutrients, which primarily supply energy, these compounds directly regulate molecular processes that determine the efficiency and quality of tissue regeneration.

Vitamins A and D also contribute to the regulation of postoperative inflammation and tissue repair. Vitamin A supports epithelial barrier integrity, mucosal immunity, and immune cell differentiation, including T-cell responses and immunoglobulin A production, thereby influencing host–microbiome interactions. Vitamin D modulates innate and adaptive immune responses, regulates inflammatory cytokine production, and supports wound healing and tissue regeneration. Given the high prevalence of vitamin D insufficiency in many populations, inadequate vitamin D status may contribute to impaired postoperative recovery and delayed healing [86,87,88,89,90,91,92,93].

Zinc plays a central role in DNA synthesis, cell proliferation, and immune regulation. It is required for the activity of numerous transcription factors and metalloproteinases involved in epithelialization and matrix remodelling [81,86]. Zinc deficiency disrupts keratinocyte migration, impairs macrophage phagocytic capacity, and prolongs inflammatory signalling, resulting in delayed wound closure and increased susceptibility to infection [86,87].

Copper is essential for angiogenesis and connective tissue stability through its role as a cofactor for lysyl oxidase, the enzyme responsible for collagen and elastin cross-linking. Inadequate copper availability compromises neovascularization and tensile strength of healing tissues, while sufficient copper levels support organized extracellular matrix formation and vascular maturation [82,88].

Selenium contributes to immune resolution primarily through its incorporation into selenoproteins such as glutathione peroxidases and thioredoxin reductases, which regulate redox balance and protect immune cells from oxidative damage. Selenium deficiency amplifies ROS-mediated inflammasome activation and prolongs inflammatory responses, whereas adequate selenium availability supports antioxidant defences and macrophage-mediated resolution [81,89].

Conditionally essential amino acids further modulate postoperative inflammation and regeneration. Arginine serves as a substrate for nitric oxide synthase, promoting endothelial nitric oxide production, vasodilation, and microvascular perfusion. It also supports lymphocyte proliferation and collagen deposition [90,91]. Glutamine functions as a critical fuel for rapidly dividing immune cells and enterocytes, sustains intestinal barrier integrity, and replenishes intracellular glutathione pools, thereby limiting oxidative stress and systemic inflammation [92,93]. Beyond specific amino acids, adequate overall protein intake is essential for postoperative recovery, as it provides the substrates required for immune function, collagen synthesis, tissue repair, and maintenance of skeletal muscle mass. This is particularly important in older adults, who are more susceptible to rapid muscle loss during periods of immobilization and acute illness. Current clinical guidelines generally recommend protein intakes of approximately 1.2–2.0 g/kg/day in surgical and hospitalized patients, depending on age, nutritional status, and clinical condition [88,89,90,91,92,93].

Collectively, micronutrients and amino acids exert enzyme-level control over immune activation, redox balance, and tissue remodelling. Their coordinated availability is therefore a prerequisite for effective immune resolution and regenerative healing following surgical injury.

## 5. The Gut–Immune Axis in Postoperative Inflammation

Surgical stress profoundly affects gastrointestinal physiology, leading to transient gut barrier dysfunction, altered microbiome composition, and increased intestinal permeability. Surgical stress can also substantially alter the composition and function of the gut microbiome. Factors commonly associated with surgery, including antibiotic exposure, anaesthesia, analgesic medications, sleep disruption, reduced oral intake, and activation of the physiological stress response, may reduce microbial diversity and impair microbiome resilience. These changes can compromise intestinal barrier function, alter immune regulation, and increase susceptibility to opportunistic infections such as *Clostridioides difficile*. Baseline patient characteristics, including advanced age, comorbidity burden, and cumulative exposure to previous surgeries or antibiotic treatments, may further influence microbiome composition and contribute to interindividual differences in postoperative recovery. These changes facilitate translocation of microbial products such as lipopolysaccharide (LPS) into the systemic circulation, triggering Toll-like receptor–mediated inflammatory signalling and amplifying postoperative inflammation [28,29,94]. This phenomenon, often referred to as metabolic or postoperative endotoxemia, contributes to sustained cytokine release, endothelial activation, and immune dysregulation during recovery [30,94]. Importantly, gut-derived inflammatory signals act synergistically with tissue-derived DAMPs, reinforcing NF-κB activation and inflammasome priming in immune cells [37,95]. In addition to direct effects on immune signalling, gut microbiota may influence inflammatory responses through interactions with cyclooxygenase (COX) pathways. Microbiome-derived metabolites can modulate the production of prostaglandins and other lipid mediators, thereby affecting both inflammatory activation and resolution processes. Conversely, dietary factors that alter substrate availability for COX enzymes may influence the balance of pro-inflammatory and pro-resolving mediators, highlighting another mechanism linking nutrition, the gut microbiome, and postoperative recovery [94,95,96].

Dietary composition is a major determinant of gut barrier integrity and microbiome-derived immune signalling. Fermentable dietary fibers and resistant starches promote the production of short-chain fatty acids, including butyrate, which support intestinal barrier integrity and immune homeostasis. In contrast, omega-3 fatty acids and polyphenols primarily modulate gut microbial composition and activity, thereby contributing to anti-inflammatory signalling and gut health. Fermented foods may further support microbiome diversity and resilience, potentially enhancing host immune function and postoperative recovery [31,63]. Conversely, Western-style diets high in saturated fats and refined carbohydrates exacerbate dysbiosis, increase intestinal permeability, and may promote systemic inflammation [23,96]. Western-style dietary patterns are characterized not only by high intakes of saturated fats and refined carbohydrates, but also by low fiber consumption and a high intake of ultra-processed foods. These dietary features may contribute to gut microbial dysbiosis, impaired intestinal barrier function, and chronic low-grade inflammation through multiple mechanisms, including reduced production of beneficial microbial metabolites and increased exposure to food additives and other pro-inflammatory compounds. In addition, Western diets are often associated with a high omega-6 to omega-3 fatty acid ratio and low overall omega-3 intake. As omega-3 fatty acids serve as precursors of specialized pro-resolving mediators, adequate tissue omega-3 status before surgery may support a more efficient transition from inflammation to tissue repair and regeneration [23,28,29,30,31,96].

Although nutritional strategies that support gut barrier function and microbiome health may contribute to improved postoperative recovery, their effectiveness is likely influenced by the patient’s baseline metabolic and nutritional status. Many individuals undergoing surgery already present with poor dietary quality, reduced microbial diversity, or gut dysbiosis, which may limit the immediate benefits of dietary interventions. Furthermore, sustained improvements in microbiome composition and function are more likely to depend on long-term adherence to healthy dietary patterns. Therefore, nutritional support should be considered not only during the immediate postoperative period but also as part of a longer-term strategy to promote microbiome resilience, tissue repair, recovery, and overall health.

## 6. Translational Implications for Postoperative Tissue Regeneration

The molecular mechanisms described above, including the generation of specialized SPMs, may contribute to clinically relevant processes involved in postoperative tissue repair and regeneration. These include angiogenesis, collagen maturation, extracellular matrix remodeling, and ultimately scar quality. Effective resolution of inflammation supports endothelial stability, improves microvascular perfusion, and enhances fibroblast activity, all of which are important for proper tissue healing. In addition, controlled inflammatory signalling and timely resolution may promote organized extracellular matrix remodeling while limiting excessive fibrosis, which is important for achieving optimal functional and aesthetic outcomes after surgery [34,48,96]. Dietary modulation differs fundamentally from pharmacologic immunosuppression. While pharmacologic approaches often aim to suppress inflammatory pathways, nutritional strategies act by promoting the natural resolution of inflammation. This allows preservation of essential immune functions, including antimicrobial defence, while preventing excessive or prolonged inflammatory damage. Such a balanced approach is particularly important in surgical patients, where both infection control and tissue viability must be carefully maintained to ensure safe recovery and optimal healing [9,11]. Although growing evidence supports the role of nutrition in inflammation resolution and tissue repair, translating these findings into clinical practice remains challenging. Postoperative patients often experience reduced appetite, altered gastrointestinal function, limited mobility, and increased metabolic demands, which may compromise nutritional intake. In this context, optimizing nutritional status before and after surgery may represent a practical strategy to support recovery. Dietary interventions should be viewed as complementary to standard surgical and medical care and tailored to individual patient needs, comorbidities, and nutritional risk [32,33,34,72,96].

Clinical and translational studies further support the relevance of perioperative nutrition. Nutritional optimization before and after surgery has been associated with lower levels of postoperative inflammatory markers, improved wound-healing trajectories, and reduced complication rates across a range of surgical settings. These benefits have been observed in both general and specialized surgical populations, highlighting the broad applicability of nutritional interventions [32,33,72].

Although many clinical trials primarily focus on clinical outcomes, emerging mechanistic evidence provides a stronger biological basis for these observations. Together, these findings support the integration of targeted nutritional strategies into perioperative care pathways. Rather than being considered only supportive care, nutrition should be viewed as an active therapeutic component that can modulate inflammatory responses, promote tissue regeneration, and ultimately improve surgical outcomes.

## 7. Conclusions and Future Directions

Despite the promising mechanistic and experimental evidence supporting nutritional modulation of postoperative inflammation, clinical findings remain heterogeneous. Studies evaluating omega-3 fatty acids, antioxidant supplementation, and other dietary interventions have reported variable results, likely reflecting differences in study design, patient populations, nutritional status, intervention timing, dosage, and outcome measures. Furthermore, many proposed mechanisms are derived from preclinical models and have not yet been fully validated in surgical patients. Although nutritional strategies aimed at supporting inflammation resolution appear promising, the optimal composition, timing, and clinical applicability of such interventions remain incompletely defined. Further well-designed clinical trials are needed to establish their effectiveness and determine which patient populations are most likely to benefit.

The current understanding of postoperative inflammation has shifted from a predominantly cytokine-centered perspective toward a systems-level view that integrates immunology, metabolism, mitochondrial biology, and tissue regeneration. This expanded framework reveals that postoperative recovery is not solely determined by the magnitude of the inflammatory response, but by the precision and timing of its resolution. Consequently, future research and clinical strategies must move beyond generalized anti-inflammatory approaches and instead target the regulatory checkpoints that govern the transition from inflammatory activation to tissue repair.

A central priority for future investigation is the refinement of immunometabolic modulation in the perioperative setting. Increasing evidence indicates that immune cell function is tightly coupled to metabolic state, and that targeted nutritional or pharmacological interventions can reprogram immune cell behaviour at a fundamental level [40,97]. However, current clinical practice still relies on relatively nonspecific nutritional protocols that do not account for individual metabolic heterogeneity. Future approaches should therefore aim to integrate metabolic phenotyping, including assessment of substrate utilization, mitochondrial function, and systemic inflammatory markers, to enable personalized perioperative nutrition strategies. Such strategies may optimize macrophage reprogramming, enhance efferocytosis, and accelerate the transition toward reparative immunity.

Another promising direction lies in the targeted modulation of mitochondrial function and intracellular quality control. As outlined previously, mitochondria act as central hubs linking metabolism, ROS generation, and innate immune activation. Therapeutic approaches that improve mitochondrial resilience, enhance mitophagy, and reduce pathological ROS production may attenuate excessive inflammasome activation and promote resolution [41,46,49]. This includes not only pharmacological agents but also nutritional components that influence mitochondrial substrates, membrane composition, and antioxidant capacity. Future studies should aim to clarify how perioperative metabolic stress alters mitochondrial dynamics in immune cells and how these changes can be selectively corrected.

The resolution phase of inflammation represents an underexplored but highly promising therapeutic target. Unlike classical anti-inflammatory strategies, which suppress immune activation broadly, pro-resolving approaches aim to actively orchestrate the termination of inflammation while preserving host defence. Specialized pro-resolving mediators have demonstrated the capacity to enhance efferocytosis, reduce neutrophil persistence, and promote tissue regeneration without inducing immunosuppression [58,59]. Future clinical research should investigate whether perioperative modulation of lipid mediator profiles, either through dietary fatty acid composition or direct administration of pro-resolving mediators, can improve surgical outcomes and reduce complication rates.

In parallel, the role of the gut microbiome and intestinal barrier function in postoperative inflammation warrants deeper investigation. Surgical stress is frequently associated with increased intestinal permeability, microbial translocation, and systemic inflammatory activation. Microbiome-derived metabolites, particularly short-chain fatty acids, influence immune cell function, epithelial integrity, and epigenetic regulation of inflammatory genes [62,98,99]. Future strategies may include targeted prebiotic, probiotic, or synbiotic interventions aimed at preserving barrier function and modulating systemic inflammation. Importantly, these approaches must be evaluated in the context of surgical timing, antibiotic exposure, and patient-specific microbiome composition.

Although omega-3 fatty acids and nutrient-rich dietary patterns may support inflammation resolution and tissue repair, their implementation in surgical patients remains challenging. The effectiveness of these interventions may depend on baseline nutritional status, long-term dietary habits, tissue fatty acid composition, and individual metabolic variability. In addition, postoperative symptoms such as nausea, pain, reduced appetite, gastrointestinal discomfort, and dietary restrictions may limit food intake and nutrient delivery during recovery. Patients who are malnourished or metabolically compromised before surgery may respond differently than those with adequate nutritional reserves. These considerations highlight the importance of individualized nutritional support and the need for practical strategies that can be implemented in real-world clinical settings. Further research is needed to determine the optimal timing, delivery, and composition of nutritional interventions and to establish how best to integrate them into perioperative care to improve recovery outcomes.

Epigenetic regulation represents another emerging frontier. The ability of environmental and nutritional factors to modify chromatin structure and gene expression suggests that postoperative inflammatory trajectories may be partially programmable at the transcriptional level. Future research should focus on identifying epigenetic signatures associated with efficient versus impaired wound healing and determining whether these signatures can be therapeutically modulated [60,61,62,100]. This could enable the development of interventions that stabilize pro-resolving immune phenotypes and prevent chronic inflammatory states.

From a translational perspective, one of the major challenges lies in integrating these complex biological insights into clinically applicable protocols. Postoperative inflammation is influenced by numerous variables, including surgical technique, ischemia-reperfusion injury, comorbidities, nutritional status, and perioperative care pathways. Therefore, future clinical trials must adopt a systems-based design that accounts for these interacting factors. Multimodal intervention strategies, combining optimized nutrition, metabolic support, and targeted immunomodulation, are likely to be more effective than single-agent approaches.

Another key aspect is the development of reliable biomarkers that reflect not only inflammatory intensity but also resolution capacity. Current clinical markers, such as CRP and leukocyte count, provide limited insight into the underlying immunological state. Emerging candidates include markers of mitochondrial function, lipid mediator profiles, efferocytosis activity, and immunometabolic signatures. The identification and validation of such biomarkers would enable real-time monitoring of postoperative immune trajectories and facilitate individualized therapeutic interventions.

In conclusion, postoperative inflammation should be conceptualized as a resolution-dependent network in which immune signalling, metabolism, mitochondrial function, and tissue repair are tightly interconnected. The success of wound healing depends not merely on suppressing inflammation, but on enabling its controlled progression toward resolution. This paradigm shift has significant implications for both research and clinical practice. By targeting the molecular mechanisms that regulate immunometabolic adaptation, mitochondrial integrity, efferocytosis, and pro-resolving signalling, it may be possible to improve surgical outcomes, reduce complications, and enhance tissue regeneration. Future studies should evaluate the optimal timing, dosage, bioavailability, and delivery of nutritional interventions, as well as their effectiveness across different surgical populations and levels of nutritional risk. Future advances will depend on the integration of mechanistic insights with personalized, systems-oriented therapeutic strategies that align with the biological complexity of postoperative recovery.

## 8. Limitations

This review has several limitations. As a narrative review, no formal risk-of-bias assessment, evidence grading, or protocol registration was performed, and no quantitative synthesis was undertaken. Although a structured search strategy and critical appraisal were applied, study selection and interpretation remain subject to potential bias, and the absence of standardized assessment tools may limit reproducibility and comparability.

The included studies are heterogeneous in design, methodology, and experimental context, encompassing in vitro models, animal studies, observational studies and clinical investigations. Much of the evidence discussed originates from mechanistic, experimental, and preclinical studies, while direct clinical evidence in surgical patients remains limited for many of the pathways described. Consequently, the proposed associations should be interpreted as biologically plausible and hypothesis-generating rather than definitive evidence of clinical efficacy.

Clinical studies investigating dietary modulation of postoperative inflammation are often limited by variability in nutritional interventions, patient populations, surgical procedures, and outcome measures, which complicates direct comparison and synthesis. Furthermore, while associations between dietary factors and inflammatory pathways are well supported, causal relationships in the clinical postoperative setting remain incompletely defined.

An important consideration is the substantial heterogeneity among surgical patients. The magnitude and duration of postoperative inflammation may vary according to factors such as age, nutritional and metabolic status, obesity, frailty, underlying disease, type of surgery, and perioperative medication exposure. These variables may also influence responsiveness to nutritional interventions and the capacity for inflammation resolution and tissue repair. Consequently, nutritional strategies that are effective in one patient population may not produce the same effects in another. Future studies should further explore these sources of variability and support the development of more individualized perioperative nutritional approaches.

An additional challenge in translating nutritional strategies into clinical practice relates to the bioavailability and pharmacokinetic properties of many bioactive compounds. Several polyphenols exhibit limited oral bioavailability, extensive metabolism, and variable tissue distribution, which may reduce their biological effects in vivo. Similarly, the production and activity of specialized pro-resolving mediators are influenced by complex metabolic processes and individual variability. These factors may partly explain the discrepancy between promising mechanistic findings and inconsistent clinical results. Further research is needed to clarify how absorption, metabolism, and tissue availability influence the effectiveness of nutritional interventions in surgical patients.

Finally, the integration of immunological, metabolic, and nutritional data remains an evolving field, and important aspects, particularly related to immunometabolic heterogeneity, mitochondrial function, microbiome interactions, and inflammation resolution biology, require further investigation. Well-designed clinical studies are needed to validate many of the mechanisms discussed and to support the development of targeted, evidence-based, and personalized perioperative nutritional strategies.

## Data Availability

No new data were created or analyzed in this study. Data sharing is not applicable to this article.

## Abbreviations

The following abbreviations are used in this manuscript:

AA	Arachidonic Acid
AMPK	AMP-Activated Protein Kinase
ATP	Adenosine Triphosphate
COX	Cyclooxygenase
DAMPs	Damage-Associated Molecular Patterns
DHA	Docosahexaenoic Acid
DNA	Deoxyribonucleic Acid
EPA	Eicosapentaenoic Acid
FFAR4	Free Fatty Acid Receptor 4
GPCR	G-Protein-Coupled Receptor
HMGB1	High-Mobility Group Box 1
HIF-1α	Hypoxia-Inducible Factor 1 Alpha
ICAM-1	Intercellular Adhesion Molecule 1
IL	Interleukin
iNOS	Inducible Nitric Oxide Synthase
LOX	Lipoxygenase
LPS	Lipopolysaccharide
MAPK	Mitogen-Activated Protein Kinase
mtDNA	Mitochondrial DNA
mTOR	Mechanistic Target of Rapamycin
NF-κB	Nuclear Factor Kappa B
NLRP3	NOD-, LRR- and Pyrin Domain-Containing Protein 3
Nrf2	Nuclear Factor Erythroid 2–Related Factor 2
PPAR	Peroxisome Proliferator-Activated Receptor
PUFAs	Polyunsaturated Fatty Acids
RAGE	Receptor for Advanced Glycation End Products
ROS	Reactive Oxygen Species
SPMs	Specialized Pro-Resolving Mediators
TLR	Toll-Like Receptor
TNF-α	Tumor Necrosis Factor Alpha
TRIF	TIR-Domain-Containing Adapter-Inducing Interferon-β
VCAM-1	Vascular Cell Adhesion Molecule 1
VEGF	Vascular Endothelial Growth Factor
